# Supplementary material for: Tissue specificity and chromosomal alterations shape divergent immune programs in HRD tumors
Source: bioRxiv. 2025 Oct 14:2025.10.13.681986. Preprint. [Version 1] doi: 10.1101/2025.10.13.681986 (PMC12633024; doi:10.1101/2025.10.13.681986)
Supplement: 1 [file NIHPP2025.10.13.681986v1-supplement-1.pdf]

bioRxiv preprint doi: https://doi.org/10.1101/2025.10.13.681986; this version posted October 14, 2025. The copyright holder for this preprint (which was not certified by peer review) is the author/funder, who has granted bioRxiv a license to display the preprint in perpetuity. It is made available under aCC-BY 4.0 International license.

Supplementary Figures

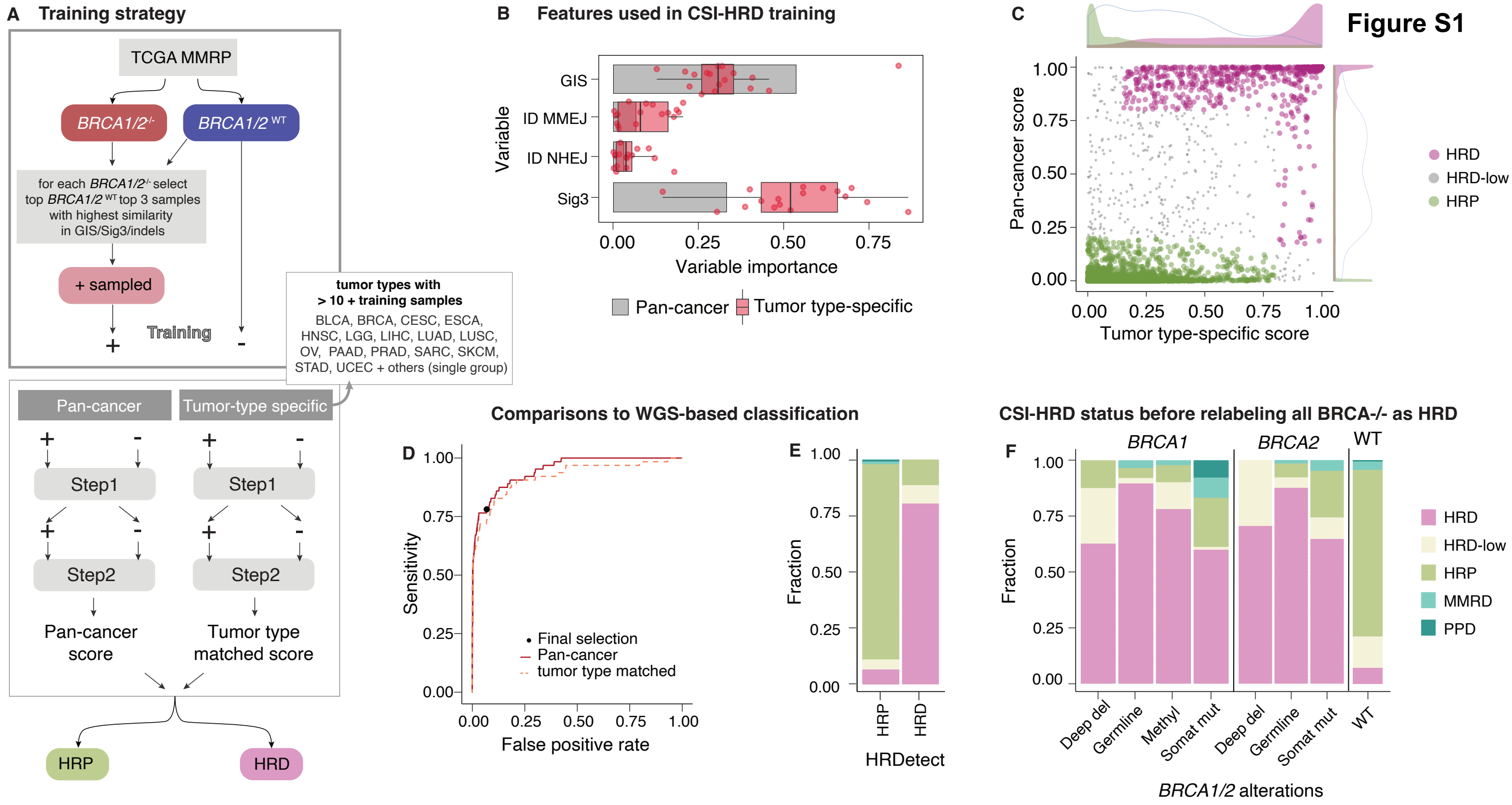
Figure S1
A Training strategy
TCGA MMRP
BRCA1/2-/-
BRCA1/2 WT
for each BRCA1/2-/- select top BRCA1/2 WT top 3 samples with highest similarity in GIS/Sig3/indels
+ sampled
Training
+
-
tumor types with > 10 + training samples
BLCA, BRCA, CESC, ESCA, HNSC, LGG, LIHC, LUAD, LUSC, OV, PAAD, PRAD, SARC, SKCM, STAD, UCEC + others (single group)
Pan-cancer
Tumor-type specific
Step1
Step2
Pan-cancer score
Tumor type matched score
HRP
HRD
B Features used in CSI-HRD training
GIS
ID MMEJ
ID NHEJ
Sig3
Variable
Variable importance
0.00
0.25
0.50
0.75
Pan-cancer
Tumor type-specific
C
Pan-cancer score
Tumor type-specific score
0.00
0.25
0.50
0.75
1.00
HRD
HRD-low
HRP
Comparisons to WGS-based classification
D
Sensitivity
False positive rate
Final selection
Pan-cancer
tumor type matched
E
Fraction
HRP
HRD
HRDetect
CSI-HRD status before relabeling all BRCA-/- as HRD
F
BRCA1
BRCA2
WT
Fraction
Deep del
Germline
Methyl
Somat mut
Deep del
Germline
Somat mut
WT
BRCA1/2 alterations
HRD
HRD-low
HRP
MMRD
PPD

bioRxiv preprint doi: https://doi.org/10.1101/2025.10.13.681986; this version posted October 14, 2025. The copyright holder for this preprint (which was not certified by peer review) is the author/funder, who has granted bioRxiv a license to display the preprint in perpetuity. It is made available under aCC-BY 4.0 International license.

Figure S1. CSI-HRD classification strategy and performance

(A) Steps of the training procedure for the CSI-HRD training. As the first step, *BRCA1/2*$^{-/-}$ samples were identified and used to select additional *BRCA1/2*^{WT} samples with matching Sig3, GIS, and indel profiles (three top matches picked for each *BRCA1/2*$^{-/-}$ sample, with replacement, increasing the positive set overall by a factor of 2.3), while the rest of the samples were used as negatives. Two approaches were followed for training: (i) pan-cancer and (ii) tumor type-specific (for types with more than 10 positive samples, as listed). For the remaining tissues, all samples were combined. Both approaches followed a two-step procedure, with an initial classification and retraining using updated labels—mainly to account for additional HRD samples that may have been left in the negative group.

(B) Variable importance of the four features that were combined into a multivariate score by the gradient boosting classifier. The bar plot shows values for the pan-cancer model; boxplots and data points indicate individual tumor type-specific classifiers.

(C) Scatter plot showing CSI-HRD scores for pan-cancer and tumor type-specific classifiers. Colors indicate final categories (MMRD and PPD samples have been excluded). The density distributions on the top and right show projections of the scores on the two axes: filled colors represent HRD and HRP groups, and the HRD-low group is shown with a grey line.

(D) Receiver operating characteristic (ROC) curves for pan-cancer and tumor type-specific scores (merging all tumor types). The black data point reflects the sensitivity and false positive rate for the selection outlined in panel C.

(E) Fraction of samples in HRP, HRD, HRD-low, MMRD, and PPD categories—based on CSI-HRD from WES data—is shown within HRD and HRP groups defined by HRDetect WGS classification.

(F) Fraction of HRP, HRD, HRD-low, MMRD, and PPD samples in BRCA1/2$^{-/-}$ (subdivided by germline, somatic, deep deletion, or promoter hypermethylation events) versus BRCA1/2^{WT} samples.

Figure S2

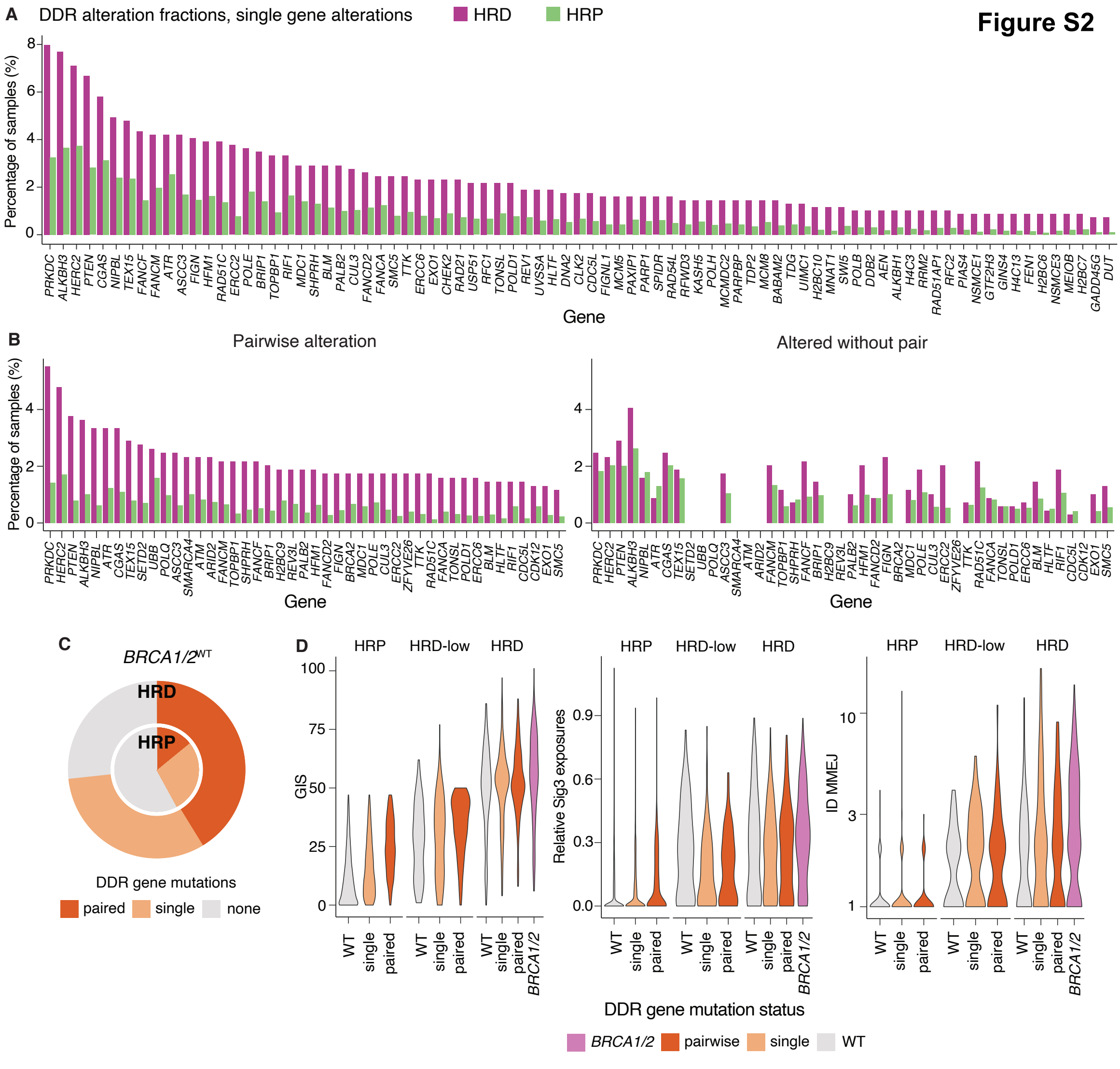
A
DDR alteration fractions, single gene alterations
HRD
HRP
Percentage of samples (%)
0
2
4
6
8
PRKDC ALKBH3 HERC2 PTEN CGAS NIPBL TEX15 FANCF FANCM ATR ASCC3 FIGN HFM1 RAD51C ERCC2 POLE BRIP1 TOPBP1 RIF1 MDC1 SHPRH BLM PALB2 CUL3 FANCD2 FANCA SMC5 TTK ERCC6 EXO1 CHEK2 RAD21 USP51 RFC1 TONSL POLD1 REV1 UVSSA HLTF DNA2 CLK2 CDC5L FIGNL1 MCM5 PAXIP1 PARP1 SPIDR RAD54L RFWD3 KASH5 POLH MCMDC2 PARPBP TDP2 MCM8 BABAM2 TDG UIMC1 H2BC10 MNAT1 SWI5 POLB DDB2 AEN ALKBH1 H4C3 RRM2 RAD51AP1 RFC2 PIAS4 NSMCE1 GTF2H3 GINS4 H4C13 FEN1 H2BC6 NSMCE3 MEIOB H2BC7 GADD45G DUT
Gene
B
Pairwise alteration
Altered without pair
Percentage of samples (%)
0
2
4
PRKDC HERC2 PTEN ALKBH3 NIPBL ATR CGAS TEX15 SETD2 UBB POLQ ASCC3 SMARCA4 ATM ARID2 FANCM TOPBP1 SHPRH FANCF BRIP1 H2BC9 REV3L PALB2 HFM1 FANCD2 FIGN BRCA2 MDC1 POLE CUL3 ERCC2 ZFYVE26 TTK RAD51C FANCA TONSL POLD1 ERCC6 BLM HLTF RIF1 CDC5L CDK12 EXO1 SMC5
Gene
C
BRCA1/2WT
HRD
HRP
DDR gene mutations
paired
single
none
D
HRP
HRD-low
HRD
GIS
0
25
50
75
100
Relative Sig3 exposures
0.0
0.3
0.6
0.9
ID MMEJ
1
3
10
WT
single
paired
BRCA1/2
DDR gene mutation status
BRCA1/2
pairwise
single
WT

bioRxiv preprint doi: https://doi.org/10.1101/2025.10.13.681986; this version posted October 14, 2025. The copyright holder for this preprint (which was not certified by peer review) is the author/funder, who has granted bioRxiv a license to display the preprint in perpetuity. It is made available under aCC-BY 4.0 International license.

Figure S2. DNA damage repair (DDR) gene alterations enriched in HRD samples

(A) Samples with damaging mutations, deep deletions, promoter hypermethylation, or biallelic loss (due mutations + LOH, two mutations, deep deletion, or promoter hypermethylation) in DDR genes (Table S2) were identified. The prevalence of these alterations was compared (comparisons done for each alteration type) between HRD and HRP samples, and the bar plot shows the percentage of samples for genes with significant enrichment (all mutation types with significant enrichment combined into a broad alteration status).

B) Instead of requiring only one DDR alteration, samples with a TP53-co-occurring pair of alterations (i.e., both present) were considered. The fraction of samples with a mutation in the presence of a significantly enriched pair (left), and those without such a pair (right), is shown.

(C) Pie chart comparing the fraction of HRD and HRP samples with DDR alterations, further stratified based on whether they appear as pairs or as single alterations.

(D) From left to right, the distributions of GIS, relative Sig3 exposure (exposure divided by total SNV counts), and ID counts at microhomologies are shown for samples stratified based on DDR alteration status and HRD status.

Figure S3

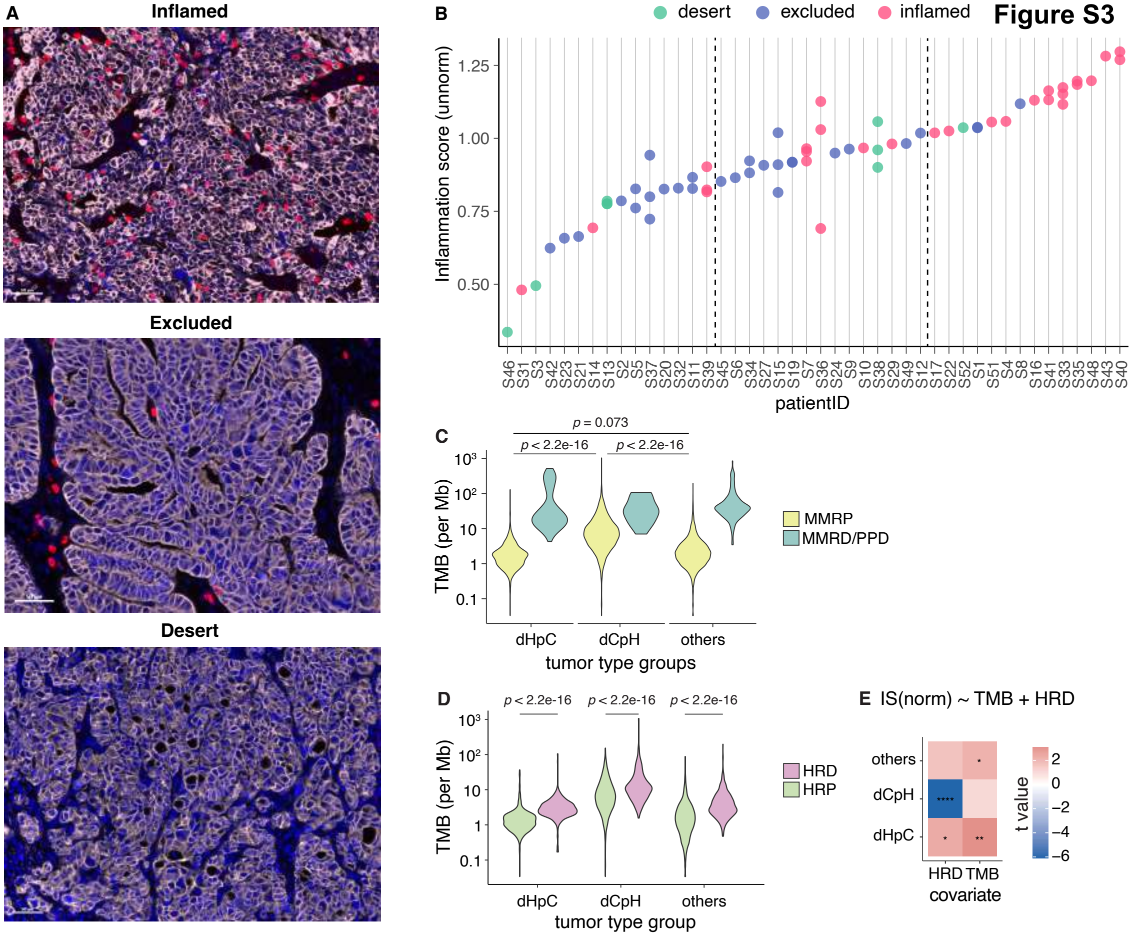

A
Inflamed
Excluded
Desert
B
desert
excluded
inflamed
Inflammation score (unnorm)
1.25
1.00
0.75
0.50
S46 S31 S3 S42 S23 S21 S14 S13 S2 S5 S37 S20 S32 S11 S39 S45 S6 S34 S27 S15 S19 S7 S36 S24 S9 S10 S38 S29 S49 S12 S17 S22 S52 S1 S51 S4 S8 S16 S41 S33 S35 S48 S43 S40
patientID
C
p = 0.073
p < 2.2e-16
p < 2.2e-16
TMB (per Mb)
10^3
10^2
10
1
0.1
dHpC
dCpH
others
tumor type groups
MMRP
MMRD/PPD
D
p < 2.2e-16
p < 2.2e-16
p < 2.2e-16
TMB (per Mb)
10^3
10^2
10
1
0.1
dHpC
dCpH
others
tumor type group
HRD
HRP
E IS(norm) ~ TMB + HRD
others
dCpH
dHpC
*
****
*
**
HRD
TMB
covariate
t value
2
0
−2
−4
−6

bioRxiv preprint doi: https://doi.org/10.1101/2025.10.13.681986; this version posted October 14, 2025. The copyright holder for this preprint (which was not certified by peer review) is the author/funder, who has granted bioRxiv a license to display the preprint in perpetuity. It is made available under aCC-BY 4.0 International license.

Figure S3. Validating the inflammation score and assessing the confounding effect of TMB

(A) Example multiplex immunofluorescence from our treatment-naïve independent cohort of immune-classified ovarian cancers showing representative cases of inflamed, excluded, and desert tumors[10].

(B) The inflammation score in our independent cohort (see panel A) showed a higher abundance of inflamed tumors in the top tertile and excluded tumors in the bottom tertile. The tertiles are separated with a dashed line. For some patients, multiple samples were sequenced with bulk RNA-seq, and due to intratumor heterogeneity, there are differences across samples from a single patient. Note that in patients with high heterogeneity, the mIF-based classification and bulk RNA-seq may capture different tumor populations and show higher disagreement.

(C) TMB distribution between MMRP (including subgroups HRP, HRD, and HRD-low) samples in dHpC, dCpH, and other tumor types is compared (t-test; p-values shown). The MMRP samples are shown separately.

(D) TMB distributions between HRD and HRP samples are compared within each tumor type group (dHpC, dCpH and others).

(E) T values (as indicated by fill colors) obtained from the multi-linear model for Z-normalized IS using HRD status and TMB as covariates performed on groups of tumor types (dHpC, dCpH and others). *p: 0.05-0.01, 0.01-0.001, 0.001-0.0001, and* < *0.0001* are indicated by '.', '*', '**', '***', and '****'.

Figure S4

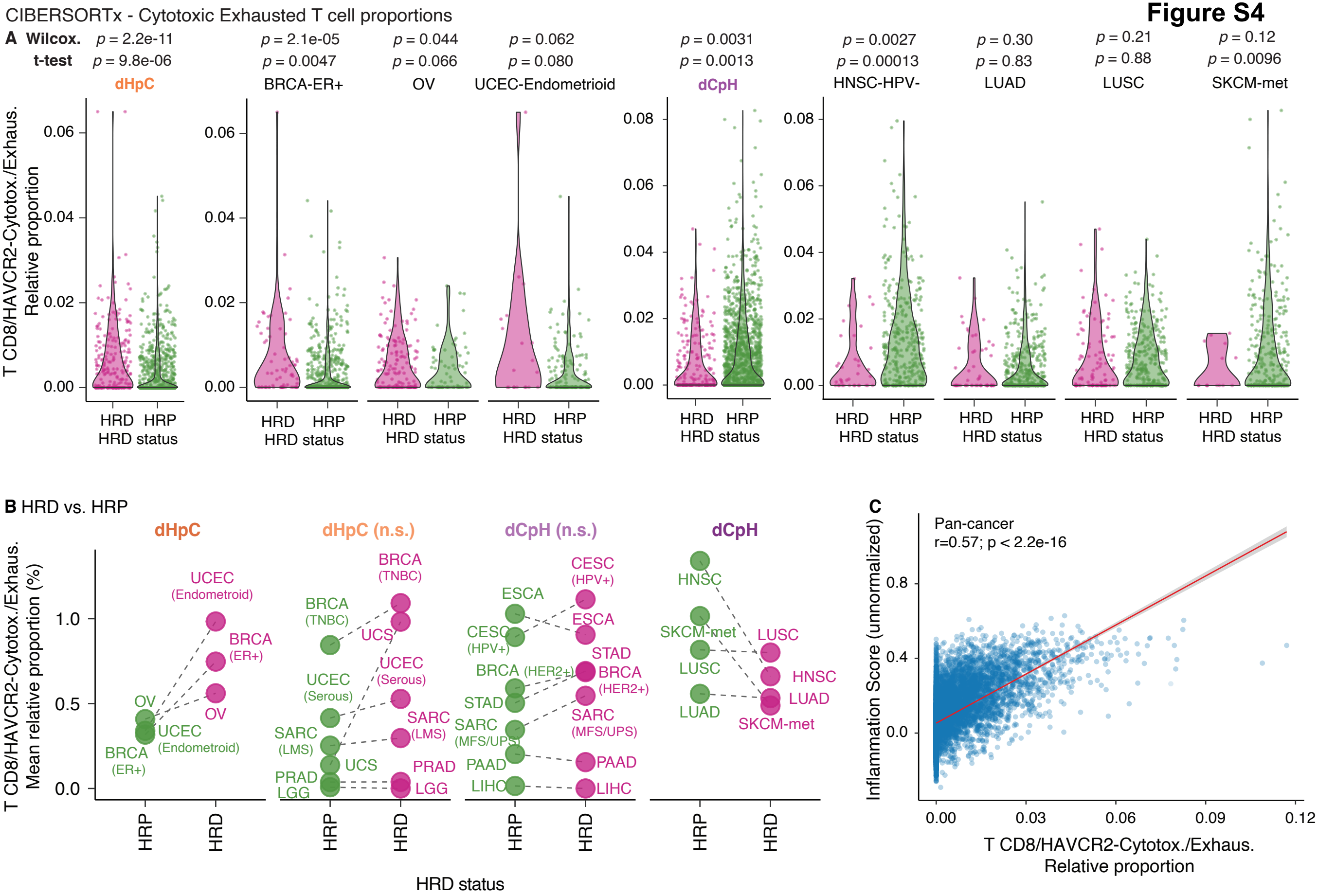
CIBERSORTx - Cytotoxic Exhausted T cell proportions
A Wilcox. p = 2.2e-11 p = 2.1e-05 p = 0.044 p = 0.062 p = 0.0031 p = 0.0027 p = 0.30 p = 0.21 p = 0.12
t-test p = 9.8e-06 p = 0.0047 p = 0.066 p = 0.080 p = 0.0013 p = 0.00013 p = 0.83 p = 0.88 p = 0.0096
dHpC
BRCA-ER+
OV
UCEC-Endometrioid
dCpH
HNSC-HPV-
LUAD
LUSC
SKCM-met
T CD8/HAVCR2-Cytotox./Exhaus.
Relative proportion
HRD HRP
HRD status
B HRD vs. HRP
dHpC
dHpC (n.s.)
dCpH (n.s.)
dCpH
T CD8/HAVCR2-Cytotox./Exhaus.
Mean relative proportion (%)
UCEC (Endometroid)
BRCA (ER+)
OV
BRCA (TNBC)
UCS
UCEC (Serous)
SARC (LMS)
PRAD
LGG
CESC (HPV+)
ESCA
STAD
BRCA (HER2+)
SARC (MFS/UPS)
PAAD
LIHC
HNSC
SKCM-met
LUSC
LUAD
HRP
HRD
HRD status
C
Pan-cancer
r=0.57; p < 2.2e-16
Inflammation Score (unnormalized)
T CD8/HAVCR2-Cytotox./Exhaus.
Relative proportion

bioRxiv preprint doi: https://doi.org/10.1101/2025.10.13.681986; this version posted October 14, 2025. The copyright holder for this preprint (which was not certified by peer review) is the author/funder, who has granted bioRxiv a license to display the preprint in perpetuity. It is made available under aCC-BY 4.0 International license.

Figure S4. Cytotoxic Exhausted CD8+ T cells across tumor types and correlations with IS.
(A) The distribution of relative proportions of cytotoxic/exhausted HAVCR2$^+$ CD8$^+$ T cells as inferred by CIBERSORTx. Proportions in HRD and HRP tumors are compared by the Wilcoxon test and t-test (*p* values are shown on top) for dHpC and dCpH groups combined and individual tumor types in these groups.
(B) The tumor type-averaged values of the HAVCR2$^+$ CD8$^+$ T in dHpC, dHpC-n.s., dCpH-n.s., and dCpH.
(C) Correlation between inflammation score (no tumor type-specific Z-score normalization) and HAVCR2$^+$ CD8$^+$ T cell relative proportions. Pearson correlation test was performed.

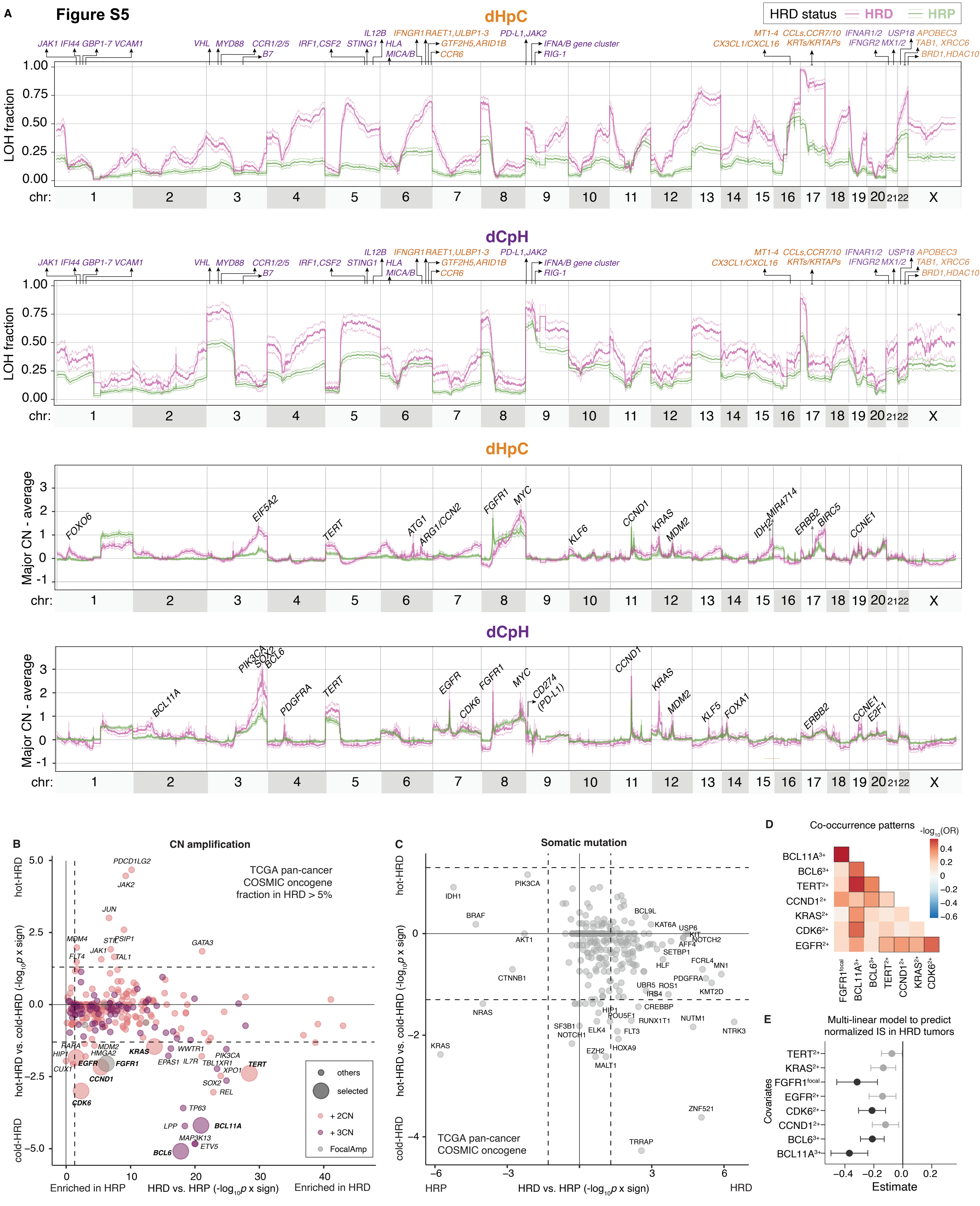

A
Figure S5
dHpC
HRD status HRD HRP
LOH fraction
chr:
dCpH
Major CN - average
B
CN amplification
TCGA pan-cancer
COSMIC oncogene
fraction in HRD > 5%
hot-HRD
cold-HRD
hot-HRD vs. cold-HRD (-log10p x sign)
Enriched in HRP
HRD vs. HRP (-log10p x sign)
Enriched in HRD
others
selected
+ 2CN
+ 3CN
FocalAmp
C
Somatic mutation
TCGA pan-cancer
COSMIC oncogene
HRP
HRD
D
Co-occurrence patterns
-log10(OR)
E
Multi-linear model to predict
normalized IS in HRD tumors
Covariates
Estimate

bioRxiv preprint doi: https://doi.org/10.1101/2025.10.13.681986; this version posted October 14, 2025. The copyright holder for this preprint (which was not certified by peer review) is the author/funder, who has granted bioRxiv a license to display the preprint in perpetuity. It is made available under aCC-BY 4.0 International license.

Figure S5. Copy number alteration profiles and their differences between HRD and HRP tumors.
(A) Same as in Figure 4 but comparing HRD and HRP tumors from top to bottom: (i) Fraction of samples with LOH in dHpC (i) and dCpH (ii), amplification profiles as defined by major CN (after subtracting mean major CN across chromosomes) in dHpC (iii) and dCpH (iv).
(B) COSMIC oncogenes with amplifications (considering 2+, 3+ for major CN with respect to the baseline and focal amplifications; indicated by marker colors) in more than 5% of pan-cancer HRD samples were identified. The frequencies were compared between (i) hot HRD vs. cold HRD tumors, and (ii) HRD vs. HRP tumors were compared. The figure shows the scatter plot for $-\log_{10}$p-values scaled by the sign of Log-Odds Ratio (LOR) from the two comparisons. The genes enriched in HRD and cold HRD tumors were selected, in regions with multiple oncogene amplifications, the one with higher significance in hot vs cold HRD comparison was picked (*e.g.* BCL6, PIK3CA, and SOX2 are all located nearby at chr3 q26.33-q27 loci and often co-amplified). The ones selected are marked with larger marker sizes.
(C) Same as in panel B but showing oncogenic mutations.

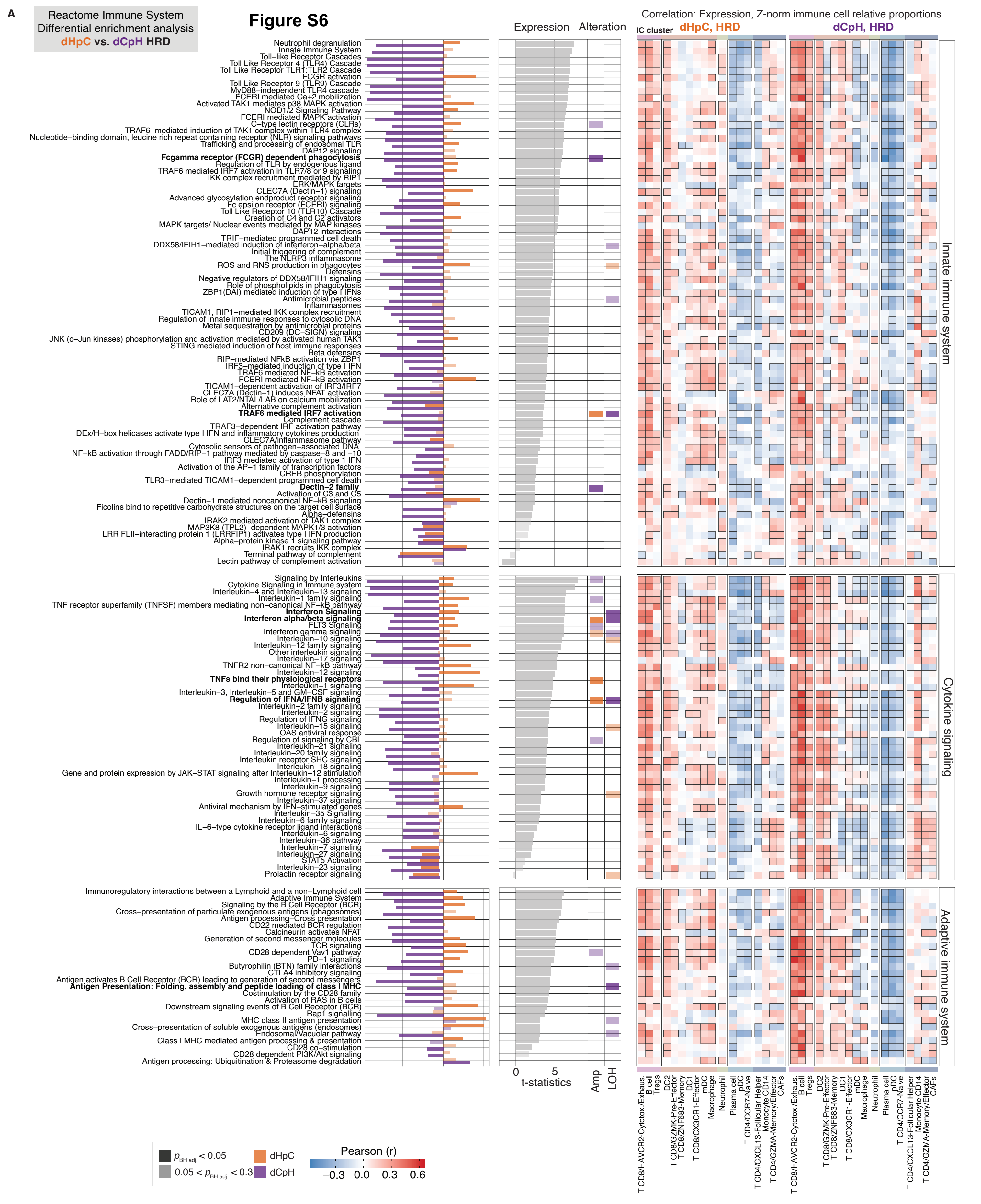

Figure S6
A
Reactome Immune System
Differential enrichment analysis
dHpC vs. dCpH HRD
Neutrophil degranulation
Innate Immune System
Toll−like Receptor Cascades
Toll Like Receptor 4 (TLR4) Cascade
Toll Like Receptor TLR1:TLR2 Cascade
FCGR activation
Toll Like Receptor 9 (TLR9) Cascade
MyD88−independent TLR4 cascade
FCERI mediated Ca+2 mobilization
Activated TAK1 mediates p38 MAPK activation
NOD1/2 Signaling Pathway
FCERI mediated MAPK activation
C−type lectin receptors (CLRs)
TRAF6−mediated induction of TAK1 complex within TLR4 complex
Nucleotide−binding domain, leucine rich repeat containing receptor (NLR) signaling pathways
Trafficking and processing of endosomal TLR
DAP12 signaling
Fcgamma receptor (FCGR) dependent phagocytosis
Regulation of TLR by endogenous ligand
TRAF6 mediated IRF7 activation in TLR7/8 or 9 signaling
IKK complex recruitment mediated by RIP1
ERK/MAPK targets
CLEC7A (Dectin−1) signaling
Advanced glycosylation endproduct receptor signaling
Fc epsilon receptor (FCERI) signaling
Toll Like Receptor 10 (TLR10) Cascade
Creation of C4 and C2 activators
MAPK targets/ Nuclear events mediated by MAP kinases
DAP12 interactions
TRIF−mediated programmed cell death
DDX58/IFIH1−mediated induction of interferon−alpha/beta
Initial triggering of complement
The NLRP3 inflammasome
ROS and RNS production in phagocytes
Defensins
Negative regulators of DDX58/IFIH1 signaling
Role of phospholipids in phagocytosis
ZBP1(DAI) mediated induction of type I IFNs
Antimicrobial peptides
Inflammasomes
TICAM1, RIP1−mediated IKK complex recruitment
Regulation of innate immune responses to cytosolic DNA
Metal sequestration by antimicrobial proteins
CD209 (DC−SIGN) signaling
JNK (c−Jun kinases) phosphorylation and activation mediated by activated human TAK1
STING mediated induction of host immune responses
Beta defensins
RIP−mediated NFkB activation via ZBP1
IRF3−mediated induction of type I IFN
TRAF6 mediated NF−kB activation
FCERI mediated NF−kB activation
TICAM1−dependent activation of IRF3/IRF7
CLEC7A (Dectin−1) induces NFAT activation
Role of LAT2/NTAL/LAB on calcium mobilization
Alternative complement activation
TRAF6 mediated IRF7 activation
Complement cascade
TRAF3−dependent IRF activation pathway
DEx/H−box helicases activate type I IFN and inflammatory cytokines production
CLEC7A/inflammasome pathway
Cytosolic sensors of pathogen−associated DNA
NF−kB activation through FADD/RIP−1 pathway mediated by caspase−8 and −10
IRF3 mediated activation of type 1 IFN
Activation of the AP−1 family of transcription factors
CREB phosphorylation
TLR3−mediated TICAM1−dependent programmed cell death
Dectin−2 family
Activation of C3 and C5
Dectin−1 mediated noncanonical NF−kB signaling
Ficolins bind to repetitive carbohydrate structures on the target cell surface
Alpha−defensins
IRAK2 mediated activation of TAK1 complex
MAP3K8 (TPL2)−dependent MAPK1/3 activation
LRR FLII−interacting protein 1 (LRRFIP1) activates type I IFN production
Alpha−protein kinase 1 signaling pathway
IRAK1 recruits IKK complex
Terminal pathway of complement
Lectin pathway of complement activation
Signaling by Interleukins
Cytokine Signaling in Immune system
Interleukin−4 and Interleukin−13 signaling
Interleukin−1 family signaling
TNF receptor superfamily (TNFSF) members mediating non−canonical NF−kB pathway
Interferon Signaling
Interferon alpha/beta signaling
FLT3 Signaling
Interferon gamma signaling
Interleukin−10 signaling
Interleukin−12 family signaling
Other interleukin signaling
Interleukin−17 signaling
TNFR2 non−canonical NF−kB pathway
Interleukin−12 signaling
TNFs bind their physiological receptors
Interleukin−1 signaling
Interleukin−3, Interleukin−5 and GM−CSF signaling
Regulation of IFNA/IFNB signaling
Interleukin−2 family signaling
Interleukin−2 signaling
Regulation of IFNG signaling
Interleukin−15 signaling
OAS antiviral response
Regulation of signaling by CBL
Interleukin−21 signaling
Interleukin−20 family signaling
Interleukin receptor SHC signaling
Interleukin−18 signaling
Gene and protein expression by JAK−STAT signaling after Interleukin−12 stimulation
Interleukin−1 processing
Interleukin−9 signaling
Growth hormone receptor signaling
Interleukin−37 signaling
Antiviral mechanism by IFN−stimulated genes
Interleukin−35 Signalling
Interleukin−6 family signaling
IL−6−type cytokine receptor ligand interactions
Interleukin−6 signaling
Interleukin−36 pathway
Interleukin−7 signaling
Interleukin−27 signaling
STAT5 Activation
Interleukin−23 signaling
Prolactin receptor signaling
Immunoregulatory interactions between a Lymphoid and a non−Lymphoid cell
Adaptive Immune System
Signaling by the B Cell Receptor (BCR)
Cross−presentation of particulate exogenous antigens (phagosomes)
Antigen processing−Cross presentation
CD22 mediated BCR regulation
Calcineurin activates NFAT
Generation of second messenger molecules
TCR signaling
CD28 dependent Vav1 pathway
PD−1 signaling
Butyrophilin (BTN) family interactions
CTLA4 inhibitory signaling
Antigen activates B Cell Receptor (BCR) leading to generation of second messengers
Antigen Presentation: Folding, assembly and peptide loading of class I MHC
Costimulation by the CD28 family
Activation of RAS in B cells
Downstream signaling events of B Cell Receptor (BCR)
Rap1 signalling
MHC class II antigen presentation
Cross−presentation of soluble exogenous antigens (endosomes)
Endosomal/Vacuolar pathway
Class I MHC mediated antigen processing & presentation
CD28 co−stimulation
CD28 dependent PI3K/Akt signaling
Antigen processing: Ubiquitination & Proteasome degradation
Expression
Alteration
0
5
t-statistics
Amp
LOH
Correlation: Expression, Z-norm immune cell relative proportions
IC cluster
dHpC, HRD
dCpH, HRD
Innate immune system
Cytokine signaling
Adaptive immune system
T CD8/HAVCR2-Cytotox./Exhaus.
B cell
Tregs
DC2
T CD8/GZMK-Pre-Effector
T CD8/ZNF683-Memory
DC1
T CD8/CX3CR1-Effector
mDC
Macrophage
Neutrophil
Plasma cell
pDC
T CD4/CCR7-Naive
T CD4/CXCL13-Follicular Helper
Monocyte CD14
T CD4/GZMA-Memory/Effector
CAFs
$p_{BH\ adj.} < 0.05$
$0.05 < p_{BH\ adj.} < 0.3$
dHpC
dCpH
Pearson (r)
−0.3
0.0
0.3
0.6

bioRxiv preprint doi: https://doi.org/10.1101/2025.10.13.681986; this version posted October 14, 2025. The copyright holder for this preprint (which was not certified by peer review) is the author/funder, who has granted bioRxiv a license to display the preprint in perpetuity. It is made available under aCC-BY 4.0 International license.

Figure S6. Multi-modal enrichment and correlative analysis for all Reactome immune signaling pathways.

Same as Figure 5A-F but for an extended set of pathways.

Figure S7

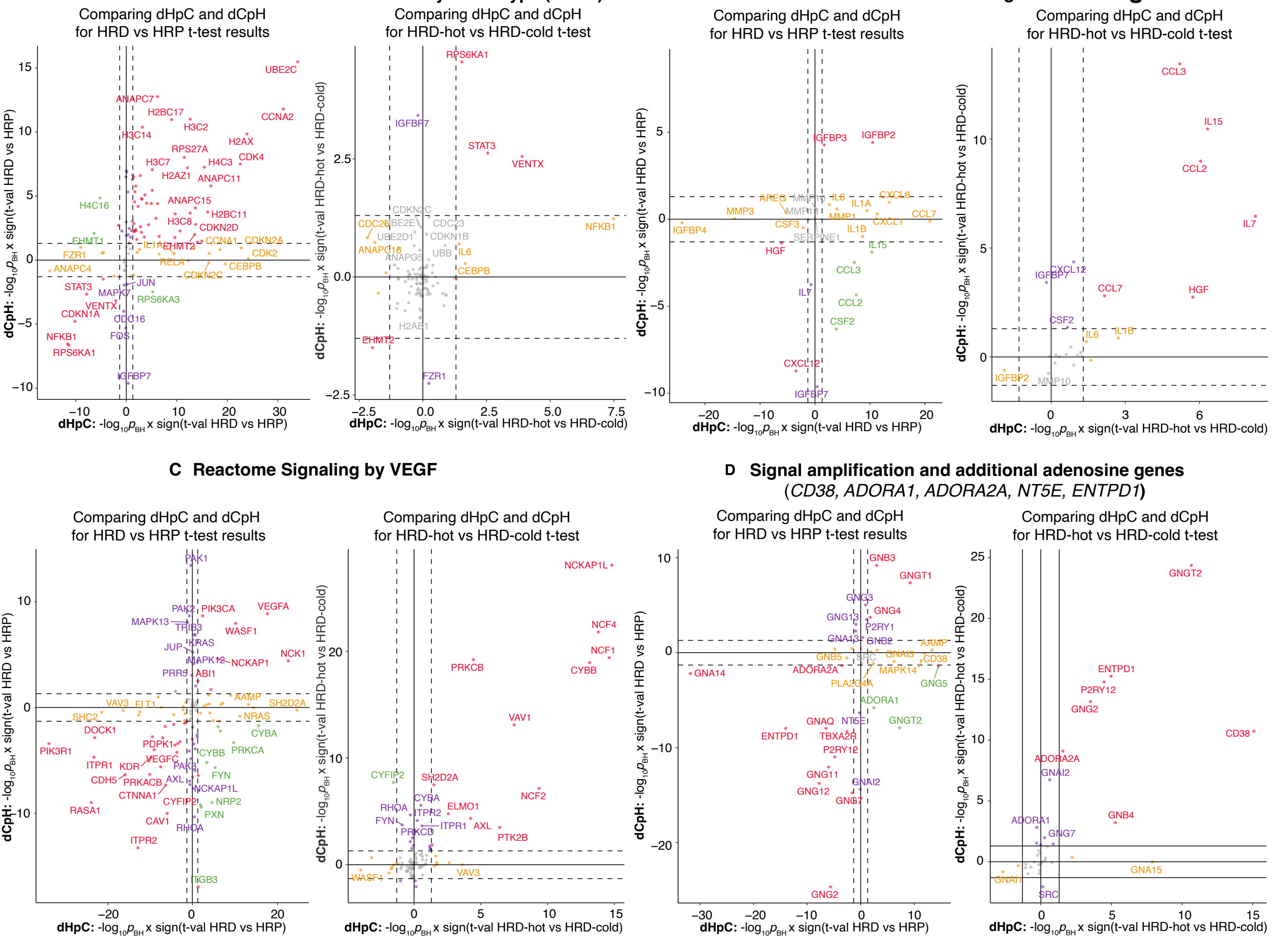
A Reactome Senescence Associated Secretory Phenotype (SASP)
Comparing dHpC and dCpH
for HRD vs HRP t-test results
Comparing dHpC and dCpH
for HRD-hot vs HRD-cold t-test
dCpH: -log10pBH x sign(t-val HRD vs HRP)
dHpC: -log10pBH x sign(t-val HRD vs HRP)
dCpH: -log10pBH x sign(t-val HRD-hot vs HRD-cold)
dHpC: -log10pBH x sign(t-val HRD-hot vs HRD-cold)
UBE2C
ANAPC7
H2BC17
CCNA2
H3C2
H3C14
H2AX
RPS27A
CDK4
H3C7
H4C3
H2AZ1
ANAPC11
H4C16
ANAPC15
H2BC11
H3C8
CDKN2D
EHMT1
IL1A
EHMT2
CCNA1
CDKN2A
FZR1
CDK2
RELA
CEBPB
ANAPC4
CDKN2C
STAT3
JUN
MAPK7
RPS6KA3
VENTX
CDKN1A
CDC16
NFKB1
FOS
RPS6KA1
IGFBP7
RPS6KA1
IGFBP7
STAT3
VENTX
CDKN2C
NFKB1
CDC26
UBE2E1
CDC23
UBE2D1
CDKN1B
ANAPC16
UBB
IL6
ANAPC5
CEBPB
H2AC1
EHMT2
FZR1
B Additional SASP genes
Comparing dHpC and dCpH
for HRD vs HRP t-test results
Comparing dHpC and dCpH
for HRD-hot vs HRD-cold t-test
IGFBP3
IGFBP2
AREG
MMP10
IL6
CXCL8
MMP3
MMP13
IL1A
CCL7
CSF3
MMP1
CXCL1
IGFBP4
IL1B
SERPINE1
HGF
IL15
CCL3
IL7
CCL2
CSF2
CXCL12
IGFBP7
CCL3
IL15
CCL2
IL7
CXCL12
IGFBP7
CCL7
HGF
CSF2
IL6
IL1B
IGFBP2
MMP10
C Reactome Signaling by VEGF
Comparing dHpC and dCpH
for HRD vs HRP t-test results
Comparing dHpC and dCpH
for HRD-hot vs HRD-cold t-test
PAK1
PAK2
PIK3CA
VEGFA
MAPK13
TRIB3
WASF1
JUP
KRAS
NCK1
MAPK12
NCKAP1
PRR5
ABI1
VAV3
FLT1
AAMP
SH2D2A
SHC2
NRAS
DOCK1
CYBA
PIK3R1
PDPK1
CYBB
PRKCA
ITPR1
KDR
VEGFC
PAK3
FYN
CDH5
PRKACB
AXL
NCKAP1L
CTNNA1
CYFIP2
NRP2
RASA1
PXN
CAV1
RHOA
ITPR2
ITGB3
NCKAP1L
NCF4
NCF1
PRKCB
CYBB
VAV1
CYFIP2
SH2D2A
CYBA
NCF2
RHOA
ITPR2
ELMO1
FYN
ITPR1
AXL
PRKCD
PTK2B
WASF1
VAV3
D Signal amplification and additional adenosine genes
(CD38, ADORA1, ADORA2A, NT5E, ENTPD1)
Comparing dHpC and dCpH
for HRD vs HRP t-test results
Comparing dHpC and dCpH
for HRD-hot vs HRD-cold t-test
GNB3
GNGT1
GNG3
GNG4
GNG13
P2RY1
GNA13
GNB2
AAMP
GNB5
SRC
GNAI3
CD38
ADORA2A
MAPK14
GNA14
PLA2G4A
GNG5
ADORA1
GNAQ
NT5E
GNGT2
ENTPD1
TBXA2R
P2RY12
GNG11
GNAI2
GNG12
GNG7
GNG2
GNGT2
ENTPD1
P2RY2
GNG2
CD38
ADORA2A
GNAI2
GNB4
ADORA1
GNG7
GNA15
GNAI1
SRC

bioRxiv preprint doi: https://doi.org/10.1101/2025.10.13.681986; this version posted October 14, 2025. The copyright holder for this preprint (which was not certified by peer review) is the author/funder, who has granted bioRxiv a license to display the preprint in perpetuity. It is made available under aCC-BY 4.0 International license.

Figure S7. Association between gene expression of selected pathways and HRD tumor archetypes. (A-D) For each panel, gene expression for genes in a given pathway was compared between HRD and HRP samples for dHpC and dCpH (left subpanels), and for HRD-hot vs HRD-cold samples in dHpC and dCpH using t-test. The x-axis shows results for dHpC in the form of $-\log_{10}p$ scaled by the sign of t value. Red indicates significant in both tumor type groups, orange in dHpC and purple in dCpH only, green indicates opposite modifications in the two tumor type groups. Results are shown in order for Reactome Senescence Associated Secretory Phenotype (SASP) pathway (R-HSA-2559582) **(A)**, additional SASP genes manually curated and not included in the reactome pathway **(B),** Reactome Signaling by VEGF (R-HSA-194138) **(C)** and signal amplification (R-HSA-392518) pathways together with additional adenosine genes (*CD38*, *ADORA1*, *ADORA2A*, *NT5E*, and *ENTPD1*) **(D)**.
